# Quantitative analysis of doxorubicin hydrochloride and arterolane maleate by mid IR spectroscopy using transmission and reflectance modes

**DOI:** 10.1186/s13065-021-00752-3

**Published:** 2021-04-24

**Authors:** Ranju Bansal, Ranjit Singh, Khushpal Kaur

**Affiliations:** University Institute of Pharmaceutical Sciences, Panjab University, Chandigarh, 160 014 India

**Keywords:** Doxorubicin hydrochloride (DOX), Arterolane maleate (ALM), Transmittance, DRIFTS

## Abstract

**Background:**

Environment-friendly fast and accurate mid-infrared spectroscopic methods have been developed for the quantitative analysis of doxorubicin hydrochloride (DOX) and arterolane maleate (ALM) in bulk and marketed formulations. Both transmittance and reflectance modes have been used for the analysis and a comparison has been drawn for better accuracy. The analytical methods were validated in accordance with International Council for Harmonisation (ICH) guidelines

**Results:**

The proposed methods have been successfully developed and validated for the quantification of doxorubicin and arterolane maleate in solid bulk and dosage form. High recovery values in both the modes, while analysing DOX and ALM, indicated good accuracy of the methods. The methods showed excellent repeatability and intermediate precision [% RSD (Relative Standard Deviation < 2.0%]. The assay values of the drugs in solid dosage forms were also found close to the labelled claim.

**Conclusion:**

The proposed Fourier transform infrared (FT-IR) spectroscopic methods were found to be specific, reproducible, valid and could be used as general methods for the quantification of most of the solid drug preparations such as tablets, capsules and powders.

**Supplementary Information:**

The online version contains supplementary material available at 10.1186/s13065-021-00752-3.

## Introduction

In recent years, Fourier transform infrared spectroscopy (FTIR) has been widely explored for the quantitative analysis, quality control, and supervision of manufacturing process of pharmaceutical products [1–3]. The different pharmaceutical preparations including liquids, solutions, pastes, powders, films, fibres, gases and surfaces can all be examined with a judicious choice of this sampling technique. FTIR is a common spectroscopic technique used by pharmaceutical chemists for the analysis of drugs because of its inherent simplicity and easy availability in most of the pharmaceutical laboratories [4, 5]. Reflectance and transmission are the two modes of FTIR spectroscopic measurements. The transmission mode is used more often in comparison to reflectance mode because it provides information of sample on its full depth and exposed cross section while the reflectance mode is good for only measuring surface properties of sample under analysis [6]. Of all the IR regions, mid-IR (MIR) region has been maximum explored for analytical purposes as this method is rapid, simple, precise, accurate and economical for the analysis of pharmaceutical preparations. Although NIR estimation is gaining popularity in pharmaceutical industry, but several drawbacks such as broadening of the absorption bands in the NIR spectra and its inability to distinguish closely related derivatives limits its usefulness [7]. The vibrational bands of MIR spectra provide better information regarding functional groups which offers broader scope to identify similar structures and presence of typical pharmaceutical excipients [8, 9].

**Fig. 1:**
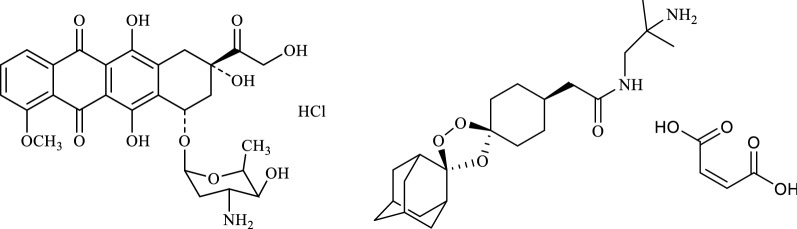
Chemical structures of doxorubicin hydrochloride and arterolane maleate

Doxorubicin hydrochloride (DOX) is a naturally occurring anthracycline derivative (Fig. 1) and is used for the treatment of a number of malignancies including solid tumors, transplantable leukemias and lymphomas [10]. It is an important component of multi-chemotherapeutic drug regimen and is usually given in combination with cyclophosphamide, vincristine, bleomycin or prednisone [10, 11]. Literature reports HPLC-UV analysis for the simultaneous determination of vincristine and doxorubicin in pharmaceutical preparations [12]. Similarly, simultaneous quantification of doxorubicin with lorazepam, metoclopramide, ondansetron, and ranitidine in IV-infusion mixtures have also been reported using LC-MS method [13]. Arnold and co-workers have developed the LC-MS/MS method to identify and quantify doxorubicin and its key metabolites in small-volume biological samples of rat plasma [14]. However, stability issues with doxorubicin such as light sensitivity and instability at high pH, in solvents and at temperature above 8 °C demands alternative analytical techniques which do not require sample preparation [15]. Use of infrared spectroscopy seems to be the most appropriate choice as it allows quantification of solid substances without using organic solvents.

Arterolane maleate (ALM) is a synthetic peroxide (Fig. 1), and is used as an anti-malarial drug [16]. It is a highly efficacious, rapidly acting blood schizonticide active against developmental stages of parasite *Plasmodium falciparum.* It is more potent in comparison with artemisinin and has a longer half-life in plasma [16, 17].

Headspace chromatographic method has been reported for the determination of residual solvents in arterolane maleate whereas liquid chromatographic methods have been predominantly used for the simultaneous estimation of ALM with piperaquine phosphate [18, 19]. Reddy and co-workers have developed and validated a stability indicating RP-HPLC method for the simultaneous estimation of arterolane maleate and piperaquine phosphate in a combined dosage form [20]. However literature lacks information regarding specific test for routine quantitative analysis of arterolane maleate in bulk. All these reported methods for quantitative estimation of doxorubicin and arterolane are expensive, time consuming, and require sample pre-treatment [12, 13, 18–20].

Accuracy and precision, the two important parameters of an analytical method, determine the closeness of the measured value to the real value and reliability of the method, respectively. To ensure adequate safety and efficacy of the drug substances, the development of simple, accurate, economical and reliable methods with high precision is the need of pharmaceutical industry for the routine quantitative analysis. Therefore, our present research work is aimed towards development of a less expensive, accurate, specific and green IR spectroscopic method for the quantitative estimation of doxorubicin hydrochloride and arterolane maleate in bulk and marketed solid drug formulations using transmittance and reflectance modes through KBr pellet and DRIFTS (Diffuse Reflectance Infrared Fourier Transform Spectroscopy) techniques. The developed methods were validated as per the ICH (International Conference Harmonization) Q2 (R1) guidelines [21].

## Materials and methods

Doxorubicin hydrochloride was obtained as a gift sample from department of nuclear sciences, Postgraduate Institute of Medical Education and Research, Chandigarh and arterolane maleate was gifted by Ranbaxy Laboratories Limited, Mohali. Marketed injection formulation of doxorubicin hydrochloride (DUXOCIN, Biochem, batch no. BND1003) and tablet formulation of arterolane maleate (SYNRIAM, Ranbaxy laboratories, batch no. 2341802) were procured from local drug store. Potassium bromide (KBr) (Uvasol quality) was purchased from Merck. All other chemicals and reagents used were of analytical grade. Infrared spectra were recorded over a spectral region from 4000 to 450 cm^−1^ using Perkin Elmer Spectrum two FT-IR spectrophotometer model accompanied with both KBr pellet and diffuse reflectance accessories in transmittance and reflectance mode, respectively. PerkinElmer version 10.03.08 Software was used for the quantification by univariate analysis. The pellets were prepared by applying 5 ton force every time using hydraulic press. The spectral resolution was kept at 8 cm^−1^ with average scans of 16 in order to obtain a good signal-to-noise ratio and these conditions were maintained throughout the experiment. Samples were measured in triplicate and for each measurement a fresh sample was prepared. The peak area ranging from 1770–1680 cm^−1^ (doxorubicin) and 1690–1630 cm^−1^ (arterolane maleate) was selected and quantified using Perkin Elmer spectrum version 10.03.08 software with baseline correction. Peak area was plotted against concentration to generate the calibration curves.

### Method A-transmission mode

#### Sample preparation

Five different concentrations (% w/w, 0.6, 0.8, 1.0, 1.2 and 1.4 in case of doxorubicin, and 0.2, 0.4, 0.6, 0.8, and 1.0 for arterolane maleate) were prepared by homogeneously mixing the required amount of the requisite drug in KBr powder to make total weight of 100 mg. A constant weight of 65 mg of sample mixture was weighed for pellet preparation. The spectra for each concentration were recorded in transmittance mode. The standard curve was obtained by plotting peak area *versus* concentration.

### Method B-reflectance mode

#### Sample preparation

Five different concentrations (% w/w) of doxorubicin and arterolane maleate were prepared in a similar way as mentioned in method A. A constant weight of 70 mg of the sample mixture was taken in a macro cup for analysis. A cover slip was then dragged across the top of the cup to remove excess powder and smooth the sample surface. The spectra obtained in reflectance mode were transformed to Kubelka Munk (KM) mathematical function. The peak area was plotted against concentration to generate the calibration curve.

### Assay validation

#### Linearity

Linearity of the proposed methods was established by analysing five different samples of the drugs (0.6%, 0.8%, 1.0%, 1.2%, 1.4% w/w of DOX and 0.2%, 0.4%, 0.6%, 0.8 %, 1.0% w/w of ALM). Least square regression method was applied for analysis of the obtained data.

#### Accuracy

To find out the accuracy of the method, three different concentrations 0.6%, 1.0%, 1.4% w/w of DOX and 0.2%, 0.6%, 1.0% w/w of ALM were prepared and analysed (*n* = 9). Accuracy was assessed as the percentage relative standard deviation (%RSD) and mean percentage recovery. Standard addition method was performed to give additional support to recovery study analysis. Specific amounts of pure drug were added to a known previously analysed concentration and the total concentration was determined using the proposed method (*n* = 3). The percent recovery of the added pure drug was calculated as,% Recovery = [(Cv – Cu)/Ca] ×100, where Cv is the total drug concentration measured after standard addition; Cu, drug concentration in the formulation; Ca, drug concentration added to formulation.

### Precision

Repeatability of the method was determined by preparing and analysing three different concentrations levels of drug concentrations 0.6%, 1.0%, 1.4% w/w of DOX and 0.2%, 0.6%, 1.0% w/w of ALM (*n* = 9), for intra and inter day precision. The triplicates were prepared for each concentration and analyzed in the same day at three different times in a day for intra-day precision study. Same protocol was followed for three different days for inter day precision study (*n* = 27). The percentage relative standard deviation was also determined.

### Limit of detection (LOD) and limit of quantitation (LOQ)

LOD and LOQ were calculated as 3.3σ/S and 10σ/S, respectively, where S is the slope of the calibration curve and σ is the standard deviation of y-intercept of regression equation.

### Robustness

Robustness of the proposed method was checked by deliberately varying the weight of the DOX-KBr and ALM-KBr mixture by ± 2 mg for estimation. Robustness was determined as percentage relative standard deviation. Three different concentrations known as LQC (low quality control), MQC (medium quality control) and HQC (high quality control) were prepared. Robustness was determined as percentage relative standard deviation.

### Estimation in formulations

Marketed lyophilized formulation ‘Duxocin®’ for doxorubicin and tablet of ‘Synriam®’ for estimation of arterolane were used. For transmission analysis, the drug (5 mg for dox and 15 mg for ALM) was weighed and mixed with KBr (95 mg for dox and 85 mg for ALM) in pestle mortar to obtain a homogenous fine powder. A pellet of 65 mg was prepared and analysed using transmission method. Likewise, five homogeneous pellets were prepared and analysed. For reflectance analysis, the macrocup was filled with 70 mg of the sample mixture and was analysed immediately to record the spectra.

## Results and discussion

### Doxorubicin hydrochloride

**Fig. 2:**
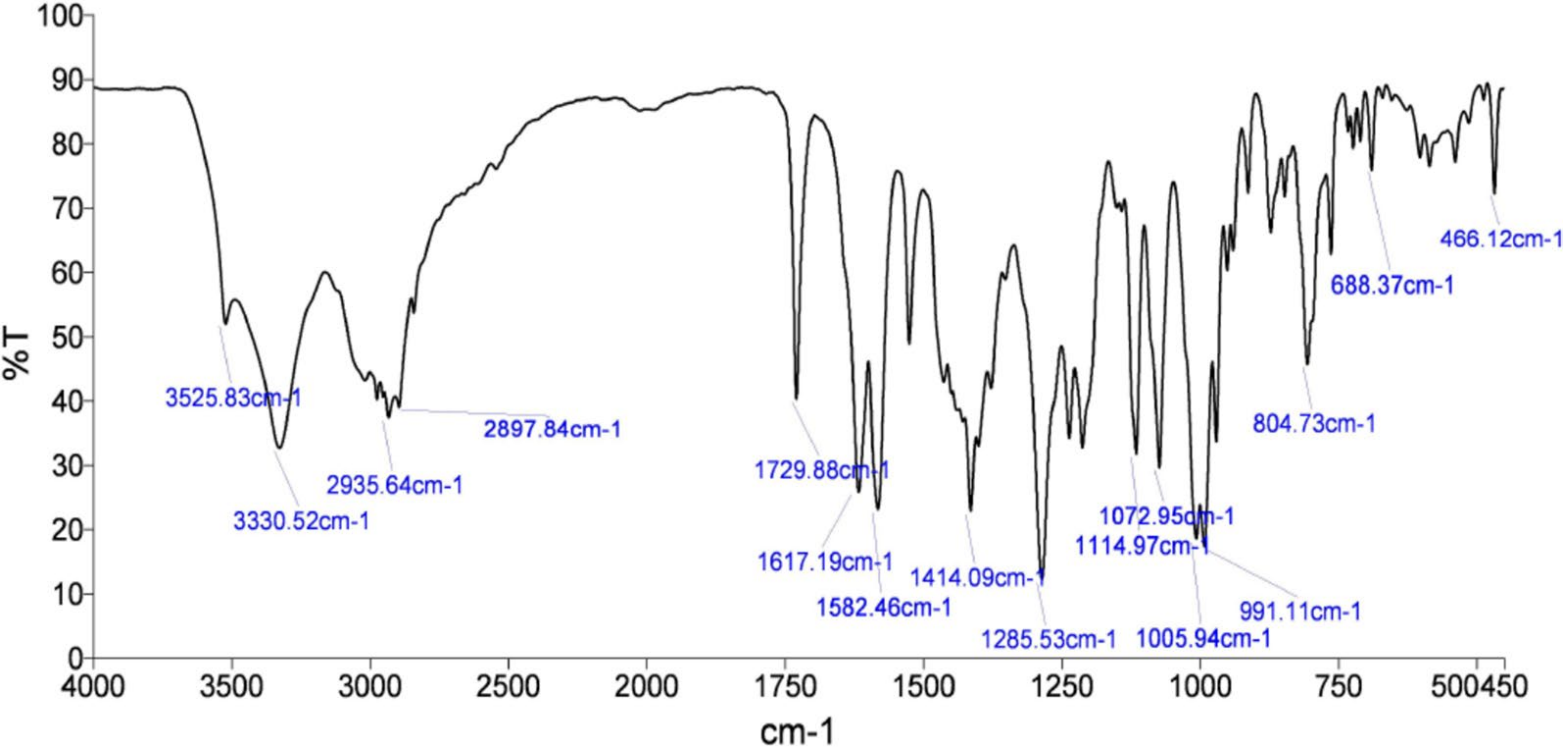
Infrared spectrum of doxorubicin hydrochloride

Several methods such as LC-MS, reverse phase HPLC and UV-Visible spectrometry have been reported and validated for quantitative analysis of doxorubicin hydrochloride in bulk and pharmaceutical dosage forms [12–14, 22, 23]. All these methods require use of solvents, however due to unstable nature of DOX at high pH, in solvents and at temperature above 8 °C, there is a need of an alternative analytical technique [15]. A fast, green and specific analytical method which do not require toxic solvents and can be performed without any sample preparation is the need of the hour. Therefore, infrared spectroscopy which does not require sample treatment provides an effective way to analyse this drug. Figure 2 depicts IR spectrum of DOX, the characteristic absorption peaks corresponding to vibrations of different functional groups of the drug molecule have been compiled in Table 1.

**Table 1:** IR spectral analysis of Doxorubicin hydrochloride

Wavenumber (cm^−1^)	Functional group
3331	N–H stretch
3525	O–H stretch
2935, 2897	C–H stretch
1729	C=O stretch
1617, 1582, 1414	C=C ring stretch
1115, 1073	C–O–C stretch
805, 688	C=H bend, C=C ring bend

### Transmittance mode versus reflectance mode

#### Standard plot

**Fig. 3:**
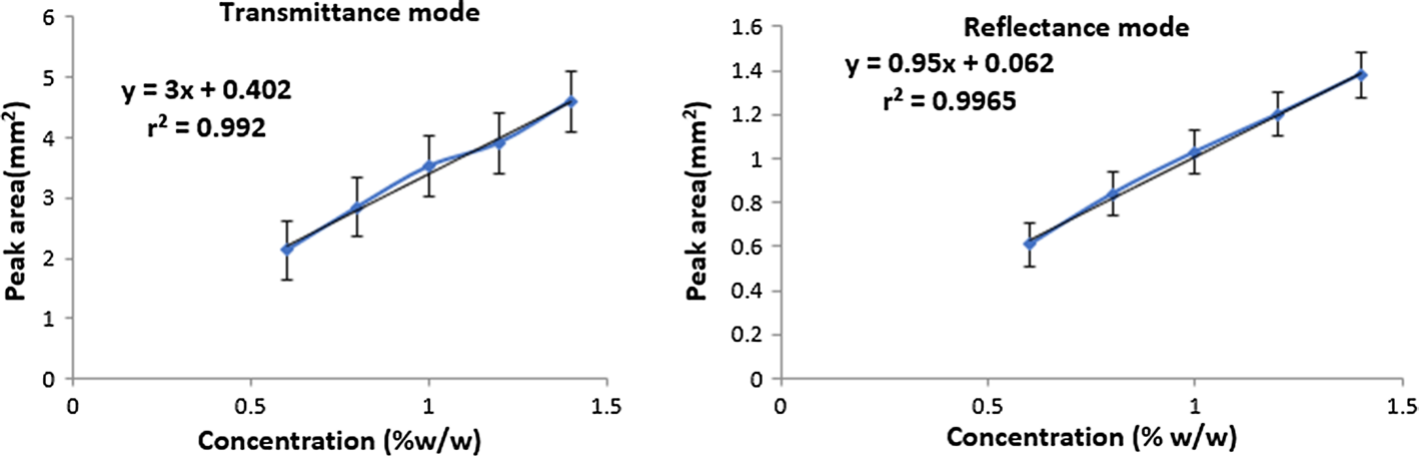
Standard plots (peak area *vs* conc.) for the selected peak (1729 cm^−1)^ of DOX in transmittance and reflectance modes

Spectra of five different concentrations (%w/w) of DOX i.e. 0.6, 0.8, 1.0, 1.2, 1.4 were taken in transmittance mode (Additional file 1: Fig. S1) and reflectance mode (Additional file 1: Fig. S2). It was noticed that transmittance from a particular concentration is also affected by the change in pellet weight (±5%), therefore, pellets were prepared from a homogenous fine powder keeping the weight constant at 65 mg. It is also noted that Lambert-Beer law is obeyed between 0.6 and 1.4% w/w in case of DOX because increasing the concentration above 1.4% w/w produced inaccurate results. The most prominent peak of DOX at 1729 cm^−1^ due to carbonyl group stretching vibrational band was selected for analytical purpose, % transmittance of which decreased in a linear fashion with increase in concentration. The calibration curve (Fig. 3) was plotted between concentration (% w/w) and peak area (mm^2^). The standard deviation (SD) values for the triplicate measurement of each concentration are given in Table 2.

**Table 2:** Calibration data of DOX quantified through transmittance and reflectance modes

Concentration (% w/w)	Transmittance mode		Reflectance mode	
	Peak area (mm^2^) *±* SD	% RSD	Peak area (mm^2^) *±* SD	% RSD
0.6	2.131 ± 0.004	0.63	0.614 ± 0.004	0.19
0.8	2.850 ± 0.005	1.30	0.843 ± 0.005	0.22
1.0	3.521 ± 0.005	0.18	1.03 ± 0.05	0.18
1.2	3.913 ± 0.003	1.17	1.201 ± 0.003	0.89
1.4	4.601 ± 0.006	0.34	1.382 ± 0.006	0.32

The spectra obtained in reflectance mode were transformed to Kubelka Munk (KM) mathematical function. In addition to the analyte composition and its proportion in the solid mixture, a DRIFTS signal may also be affected by particle size distribution, packing density, mirror alignment, spectrometer resolution, the number of scans collected and exposure to humidity. Taking all these parameters into consideration for the analysis of samples using DRIFTS technique, it was observed that with increase in concentration, % reflectance of the selected peak decreased. The calibration curve (Fig. 3) was plotted between concentration (% w/w) on the x-axis and peak area (mm^2^) on the y-axis. The standard deviation (SD) values for the triplicate measurement of each concentration are shown in Table 2.

### Method validation

Validation of an analytical procedure was done in accordance with the ICH Q2 (R1) guidelines [21] to demonstrate the suitability of the method for its intended purpose.

#### Linearity

The standard plot was linear over the concentration range 0.6–1.4% w/w. The slope, intercept and correlation coefficient values of the calibration curves of DOX in both the modes are given in Table 3. The high correlation coefficient in reflectance mode indicated better linearity over transmittance mode.

**Table 3:** Linear regression data of DOX obtained by transmittance and reflectance methods

Statistical Parameters	Transmittance mode	Reflectance mode
Concentration range	0.6% − 1.4% w/w	0.6% − 1.4% w/w
Regression equation	y = 3x + 0.402	Y = 0.95x + 0.062
Correlation coefficient	0.992	0.996
Slope	3.0	0.950
Intercept	0.402	0.062

n = 3

#### Accuracy

The accuracy of an analytical procedure expresses the closeness of agreement between the accepted reference value and the value found. The excellent mean% recovery values (nearly 100%) and their low standard deviation values (SD < 2.0) represent accuracy. The recovery studies were performed to check the accuracy of the proposed method. The mean percentage recoveries calculated for selected three different concentrations of DOX in transmittance and reflectance modes are shown in Tables 4 and 5, respectively. High recovery values ranging from 94.48 to 98.26 in transmittance mode and 92.50–99.28 in reflectance mode indicated good accuracy of the methods.

**Table 4:** Results of recovery study for DOX (Transmittance mode)

Sample drug (mg, 1:10 w/w in KBr)	Pure drug (mg, 1:10 w/w in KBr)	Total amount of drug (mg) recovered in drug-KBr mix (100 mg)	% Recovery ± SD	% RSD
5.0	1.0	0.566	94.480 ± 0.003	0.228
5.0	5.0	0.967	96.742 ± 0.002	0.385
5.0	9.0	1.375	98.261 ± 0.001	0.645

Total drug + KBr mixture = 100 mg (Pellet weight = 65 mg). n = 9

**Table 5:** Results of recovery study for DOX (reflectance mode)

Sample drug (mg, 1:10 w/w in KBr)	Pure drug (mg, 1:10 w/w in KBr)	Total amount of drug (mg) recovered in drug-KBr mix (100 mg)	% Recovery ± SD	% RSD
5.0	1.0	0.555	92.504 ± 0.002	0.228
5.0	5.0	0.969	96.943 ± 0.002	0.282
5.0	9.0	1.39	99.281 ± 0.001	0.645

Total drug + KBr mixture = 100 mg (mixture weight taken = 70 mg). n = 9

#### Precision

Precision was determined by studying repeatability and intermediate precision. Repeatability results indicate the precision under the same operating conditions over a short interval of time. Intermediate precision expresses within-laboratory variations in different days. Intermediate precision was observed for the same concentrations in triplicate on three consecutive days. The standard deviation and relative standard deviation (coefficient of variation) are reported for each type of precision investigated. Intra- and inter-day data was taken into account to determine precision of the proposed methods. The standard deviation and relative standard deviation (coefficient of variation) for the repeatability studies for the intra-day and inter-day analysis of doxorubicin hydrochloride are shown in Tables 6 and 7, respectively. The % RSD for DOX for intra-day precision was < 1.65 (Transmittance mode) and < 0.74 (Reflectance mode) as shown in Table 6. In intermediate precision study, % RSD values were not more than 1.30 in all the cases (Table 7) and were within the acceptable range indicating that this method has excellent repeatability and intermediate precision.

**Table 6:** Results of repeatability study for DOX in transmittance and reflectance modes

Concentration (% w/w)	Transmittance mode		Reflectance mode
	Disc weight (SD, % RSD)	Intra-day (n=3) (SD, % RSD)	Intra-day (n=3) (SD, % RSD)
0.6	0.87, 1.40	0.004, 1.65	0.002, 0.65
1.0	0.75, 1.17	0.003, 0.82	0.002, 0.72
1.4	0.55, 0.87	0.006, 1.14	0.003, 0.74

n = 9

**Table 7:** Results of intermediate precision study for DOX in transmittance and reflectance modes

Concentration (% w/w)	Transmittance mode		Reflectance mode
	Disc weight (SD, % RSD)	Inter-day (n=3) (SD, % RSD)	Inter-day (n=3) (SD, % RSD)
0.6	1.08, 1.72	0.002, 0.94	0.002, 0.84
1.0	0.95, 1.48	0.005, 1.30	0.004, 0.90
1.4	0.60, 0.95	0.006, 1.28	0.004, 1.08

n = 18

#### Limit of detection (LOD) and Limit of quantitation (LOQ)

The LOD and LOQ of the drug were determined using calibration standards. The LOD and LOQ values of DOX for respective analytical modes are shown in Table 8.

**Table 8:** Results of LOD and LOQ study (DOX)

Parameter	Doxorubicin hydrochloride (% w/w)	
	Transmittance mode	Reflectance mode
LOD	0.11	0.15
LOQ	0.30	0.48

n = 3

#### Robustness

Variation in weight of the DOX-KBr mixture by ± 2 mg did not produce any considerable effect on transmittance/ reflectance values. The results obtained are compiled in Table 9, which shows that small deviations in pellet or mixture weight do not affect the analytical method substantially, the reflectance mode being more robust than transmittance mode.

**Table 9:** Robustness study for DOX (transmittance and reflectance mode)

Concentration (% w/w)	Transmittance mode			Reflectance mode	
	Wt of drug-KBr mixture (mg)	Disc wt (mg)	% RSD	Wt of drug-KBr mixture (mg)	% RSD
0.6	63	62.75	4.75	68 70 72	1.68
	65	64.97			
	67	66.66			
1.0	63	62.10	3.65	68 70 72	1.70
	65	64.78			
	67	66.82			
1.4	63	63.87	4.87	68 70 72	1.46
	65	64.98			
	67	67.23			

n = 3

### Estimation in formulation

The suitability of the proposed methods was further verified by carrying out the assay on a marketed preparation (Duxocin®). The estimated drug content with low values of standard deviation (less than 4) established the precision of the proposed methods. The results obtained from the proposed methods have been shown in Table 10.

**Table 10:** Determination of DOX in formulation

Commercial product	Doxorubicin hydrochloride (10 mg)	
	Transmittance mode	Reflectance mode
Amount found (mg)^a^	9.283 ± 0.184	9.461 ± 0.198
% Purity ± SD	92.83 ± 1.58	94.61 ± 1.09

^a^Value is the mean of five estimations. n = 3

### Arterolane maleate

**Fig. 4:**
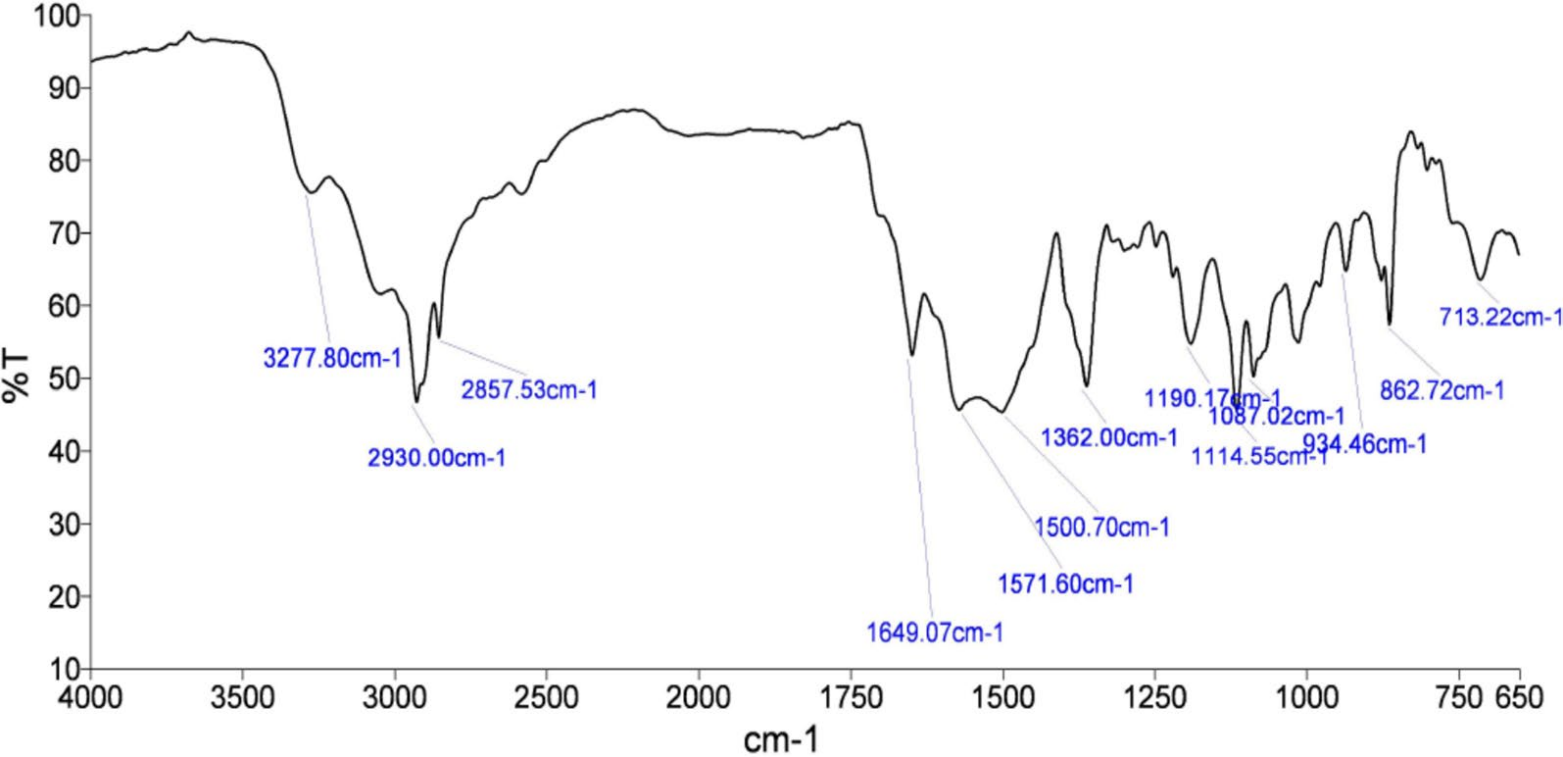
The infrared spectrum of arterolane maleate

Reverse phase HPLC methods have been used for analysis of combination of arterolane maleate and piperaquine phosphate in pharmaceutical dosage forms and a headspace gas chromatographic method for the estimation of residual solvents in ALM have been reported [17–19]. The apparent lack of a simple and highly selective method for the estimation of ALM prompted us to develop an IR based environment friendly analytical method for estimation of ALM. The proposed method was optimized and validated in accordance with the International Conference on Harmonization (ICH) guidelines. IR spectroscopic method for the determination of ALM in solid dosage form is suggested here for the first time. The characteristic absorption peaks due to various stretching and bending vibrations of arterolane maleate (Fig. 4) have been compiled in Table 11*.*

**Table 11:** IR spectral analysis of arterolane maleate

Wavenumber (cm^−1^)	Functional group
3278	N–H stretch
2930, 2858	C–H stretch
1650	Amide C=O stretch
1571, 1500	N–H bend
1362	C–H bend
1190, 1114.	C–N stretch

### Transmittance mode *versus* reflectance mode

#### Standard plot

**Fig. 5.**
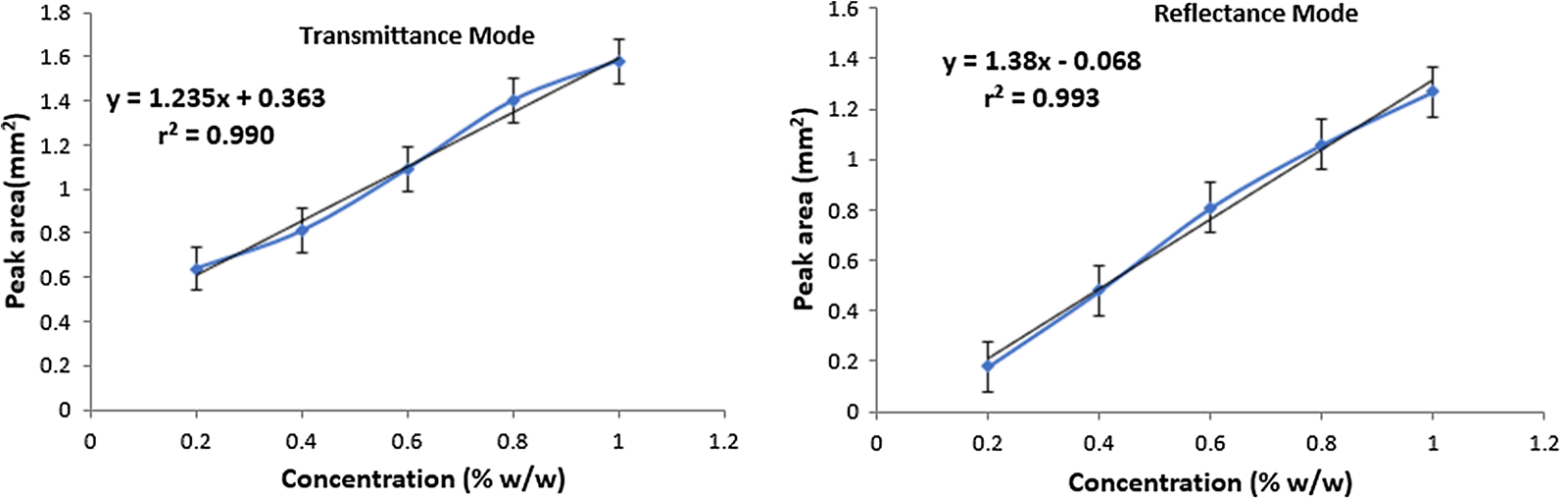
Standard plots (peak area vs conc.) for the selected peak of ALM in transmittance and reflectance modes

In a similar way, as mentioned for doxorubicin, the IR spectra of five different concentrations (% w/w, 0.2, 0.4, 0.6, 0.8, 1.0) of ALM were taken in transmittance (Additional file 1: Fig. S3) as well as reflectance modes (Additional file 1: Fig. S4). Again, a fixed weight of the prepared homogenous fine powder (65 mg) was used to prepare the pellets to avoid any changes in transmittance due to weight variations. Lambert-Beer law was obeyed between 0.2 and 1.0% w/w as any concentration out of this range gave rise to erroneous results in both the modes. The most prominent peak at 1650 cm^−1^ due to amide carbonyl stretch was selected for analysis. The linear changes in % transmittance and % reflectance of the peak at selected wavenumber (1650 cm^–1^) with changes in concentration were observed. The calibration curves for both the modes are shown in Fig.5 and the standard deviation values of the triplicate analysis for each concentration are shown in Table 12. A fixed weight of KBr-ALM mixture (70 mg) was used for analysis using reflectance mode. The spectra obtained in reflectance mode were transformed to Kubelka Munk (KM) mathematical function for quantitative analysis.

**Table 12:** Calibration data of ALM quantified through transmittance and reflectance mode

Concentration (% w/w)	Transmittance mode		Reflectance mode	
	Peak area (mm^2^) *±* SD	% RSD	Peak area (mm^2^) *±* SD	% RSD
0.2	0.644 ± 0.004	0.19	0.180 ± 0.004	0.19
0.4	0.811 ± 0.005	0.22	0.486 ± 0.005	0.15
0.6	1.091 ± 0.005	0.18	0.811 ± 0.005	0.18
0.8	1.40 ± 0.03	0.89	1.060 ± 0.003	0.89
1.0	1.581 ± 0.006	0.324	1.272 ± 0.006	0.84

### Method validation

On a similar note as for DOX, the developed method was validated according to the ICH Q2 (R1) guidelines. The data obtained from linearity, accuracy (recovery studies), precision, LOD, LOQ and robustness studies has been compiled in Tables 13, 14, 15, 16, 17, 18,  19. Calibration graph was linear over the range 0.2–1.0% w/w with reflectance mode marginally better than transmittance mode. In precision study, % RSD values were not more than 1.35% in all the observations (Tables 16 and 17) and were within the acceptable range indicating that this method has excellent repeatability and intermediate precision. Variation in weight of the ALM-KBr mixture by ± 2 mg did not have produce any significant effect on transmittance/reflectance values (Table 19). However, reflectance method was found to be more robust than transmittance method.

**Table 13:** Linear regression data of ALM obtained by transmittance and reflectance method

Statistical Parameters	Transmittance mode	Reflectance mode
Concentration range	0.2–1.0 (% w/w)	0.2–1.0 (% w/w)
Regression equation	y = 1.235x + 0.363	y = 1.38x − 0.068
Correlation coefficient	0.990	0.993
Slope	1.235	1.38
Intercept	0.363	− 0.068

n = 3

**Table 14:** Results of recovery study for ALM (transmittance mode)

Sample drug (mg, 1:20 w/w in KBr)	Pure drug (mg, 1:20 w/w in KBr)	Total amount of drug (mg) recovered in drug- KBr mix (100 mg)	% Recovery ± SD	% RSD
3.0	1.0	0.186	93.001 ± 0.003	0.450
3.0	9.0	0.55	91.660 ± 0.002	1.29
3.0	17.0	0.921	92.10 ± 0.01	0.96

Total drug + KBr mixture = 100 mg (Pellet weight = 65 mg). n = 9

**Table 15:** Results of recovery study for ALM (reflectance mode)

Sample drug (mg, 1:20 w/w in KBr)	Pure drug (mg, 1:20 w/w in KBr)	Total amount of drug (mg) recovered in drug- KBr mix (100 mg)	% Recovery ± SD	% RSD
3.0	1.0	0.182	91.00 ± 0.01	0.41
3.0	9.0	0.550	91.83 ± 0.03	1.21
3.0	17	0.919	91.90 ± 0.01	0.86

Total drug + KBr mixture = 100 mg (mixture weight taken = 70 mg). n = 9

**Table 16:** Results of repeatability study for ALM in transmittance and reflectance modes

Concentration (% w/w)	Transmittance mode		Reflectance mode
	Disc weight (SD, % RSD)	Intra-day (n=3) (SD, % RSD)	Intra-day (n=3) (SD, % RSD)
0.2	0.67, 1.23	0.002, 1.35	0.002, 0.55
0.6	0.65, 0.98	0.003, 0.72	0.005, 0.72
1.0	0.56, 0.79	0.006, 1.08	0.004, 0.82

n = 9

**Table 17:** Results of intermediate precision study of ALM in transmittance and reflectance modes

Concentration (% w/w)	Transmittance mode		Reflectance mode
	Disc weight (SD, % RSD)	Inter-day (n=3) (SD, % RSD)	Inter-day (n=3) (SD, % RSD)
0.2	0.78, 1.24	0.002, 0.89	0.002, 0.89
0.6	0.56, 1.10	0.002, 1.12	0.002, 1.03
1.0	0.60, 0.95	0.003, 1.13	0.003, 0.98

n = 18

**Table 18:** Results of LOD and LOQ study for ALM

Parameter	ALM (% w/w)	
	Transmittance mode	Reflectance mode
LOD	0.07	0.06
LOQ	0.19	0.18

n = 3

**Table 19:** Robustness study of ALM (transmittance and reflectance mode)

Concentration (% w/w)	Transmittance mode			Reflectance mode	
	Wt of drug-KBr mixture (mg)	Disc wt (mg)	% RSD	Wt of drug-KBr mixture (mg)	% RSD
0.2	63	62.91	5.98	68 70 72	1.56
	65	64.89			
	67	66.94			
0.6	63	62.10	4.86	68 70 72	1.43
	65	64.78			
	67	66.82			
1.0	63	63.87	5.48	68 70 72	1.37
	65	64.98			
	67	66.98			

n = 3

### Estimation in formulation

The suitability of the proposed method was estimated by carrying out assay of drug in marketed preparation (tablets). Assay value for ALM in tablet formulation was found to be 96.3% and 98.67% using the transmittance and reflectance modes, respectively. The estimated drug content with low values of standard deviation (less than 1.43, Table 20) established the precision of the proposed methods.

**Table 20:** Determination of ALM in tablet formulation

Commercial product	Arterolane maleate (150 mg)	
	Transmittance method	Reflectance method
Amount found (mg)^a^	144.11 ± 0.21	148.02 ± 0.12
% Purity ± SD	96.07 ± 0.12	98.68 ± 0.16

^a^Value is the mean of five estimations. n = 3

## Conclusions

The proposed methods have been successfully developed and validated for the quantification of doxorubicin and arterolane maleate in solid bulk and dosage form. The developed methods offer a simple and dependable alternative for the routine quantitative analysis of both the drugs. In this study transmittance and reflectance modes of IR spectroscopy have been compared while estimating the drugs. It was observed that a slightly better correlation coefficient (DOX, r^2^ = 0.996; ALM, r^2^ = 0.993) was observed in reflectance mode in comparison to transmittance mode (DOX, r^2^ = 0.992; ALM, r^2^ = 0.990) while analysing both the drugs. Moreover, assay values of drug substances for both drug formulations are close to the labelled claim, marginally better in case of DRIFTS method than pellet method. High recovery values ranging from 94.48 to 98.26 for DOX and 92.1–93.0 for ALM in transmittance mode, and 92.50–99.28 for DOX and 91.0–91.9 for ALM in reflectance mode indicated good accuracy of the methods. Both the methods displayed excellent repeatability and intermediate precision with % RSD values within the acceptable range (< 2%). Intra-day precision (< 0.74, DOX; < 0.82, ALM) was marginally higher in reflectance mode in comparison to transmittance mode (< 1.65, DOX; < 1.35, ALM) for both the drugs. Similar observations were made in intermediate precision study with % RSD < 1.30 in all the cases.

The developed methods are accurate, precise, robust and easy to use as compared to the time-consuming chromatographic methods reported in the literature, which also require high quantities of solvents. FTIR methods although are quite sensitive but accuracy may be affected due to manual errors, which could be minimized by practice and expertise. The proposed methods are not only free from any interference from the excipient matrix, they do not require pre-treatment of sample and are solvent free methods which could be very suitable for analysis of unstable compounds like doxorubicin hydrochloride. To conclude, for the quantitative analysis of doxorubicin hydrochloride and arterolane maleate in bulk and pharmaceutical formulation, eco-friendly FT-IR spectroscopic methods have been developed and validated as per ICH guidelines. The assay methods are found to be selective, reproducible and valid. The developed methods could be used as general-methods for the quantification of most of the solid dosage forms of the drugs such as tablets, capsules and powders.

## Supplementary Information

**Additional file 1: Figure S1.** Transmittance spectra for various concentrations (% w/w) of DOX (a) 0.6 (b) 0.8 (c) 1.0 (d) 1.2 and (e) 1.4 and % transmittance of the carbonyl peak of DOX at 1729 cm^−1^. **Figure S2.** Reflectance spectra for various concentrations of DOX (% w/w) (a) 0.6 (b) 0.8 (c) 1.0 (d) 1.2 and (e) 1.4 and the % reflectance of carbonyl peak of DOX at 1729 cm^−1^. **Figure S3.** Transmittance spectra for various concentrations (% w/w) of ALM (a) 0.2 (b) 0.4 (c) 0.6 (d) 0.8 and (e) 1.0 and the % transmittance of carbonyl peak of ALM at 1650 cm^−1^. **Figure S4.** Reflectance spectra for various concentrations (% w/w) of ALM (a) 0.2 (b) 0.4 (c) 0.6 (d) 0.8 and (e) 1.0 and the % reflectance of carbonyl peak of ALM at 1650 cm^−1^.

## Data Availability

All data generated during this study are included in this published article [and its Additional information]. Figures S1-S4 are available as Additional file. The datasets analysed during the current study available from the corresponding author on reasonable request.
