# Supplementary material for: Quantitative analysis of doxorubicin hydrochloride and arterolane maleate by mid IR spectroscopy using transmission and reflectance modes
Source: BMC Chem. 2021 Apr 24;15(1):27. doi: 10.1186/s13065-021-00752-3 (PMC8070331; doi:10.1186/s13065-021-00752-3)
Supplement: Supplementary file 1 — Additional file 1: Figure S1. Transmittance spectra for various concentrations (% w/w) of DOX (a) 0.6 (b) 0.8 (c) 1.0 (d) 1.2 and (e) 1.4 and % transmittance of the carbonyl peak of DOX at 1729 cm−1. Figure S2. Reflectance spectra for various concentrations of DOX (% w/w) (a) 0.6 (b) 0.8 (c) 1.0 (d) 1.2 and (e) 1.4 and the % reflectance of carbonyl peak of DOX at 1729 cm−1. Figure S3. Transmittance spectra for various concentrations (% w/w) of ALM (a) 0.2 (b) 0.4 (c) 0.6 (d) 0.8 and (e) 1.0 and the % transmittance of carbonyl peak of ALM at 1650 cm−1. Figure S4. Reflectance spectra for various concentrations (% w/w) of ALM (a) 0.2 (b) 0.4 (c) 0.6 (d) 0.8 and (e) 1.0 and the % reflectance of carbonyl peak of ALM at 1650 cm−1. [file 13065_2021_752_MOESM1_ESM.docx]

**SUPPLEMENTRY INFORMATION**

**Quantitative analysis of two drugs doxorubicin hydrochloride and arterolane maleate by mid IR spectroscopy using transmission and reflectance modes**

**Ranju Bansal*, Ranjit Singh, Khushpal**

University Institute of Pharmaceutical Sciences, Panjab University, Chandigarh.

**CONTENTS**

**Figure S1.** Transmittance spectra for various concentrations (% w/w) of DOX (a) 0.6 (b) 0.8 (c) 1.0 (d) 1.2 and (e) 1.4 and % transmittance of the carbonyl peak of DOX at 1729 cm^-1^

**Figure S2.** Reflectance spectra for various concentrations of DOX (% w/w) (a) 0.6 (b) 0.8 (c) 1.0 (d) 1.2 and (e) 1.4 and the % reflectance of carbonyl peak of DOX at 1729 cm^-1^.

**Figure S3.** Transmittance spectra for various concentrations (% w/w) of ALM (a) 0.2 (b) 0.4 (c) 0.6 (d) 0.8 and (e) 1.0 and the % transmittance of carbonyl peak of ALM at 1650 cm^-1^.

**Figure S4.** Reflectance spectra for various concentrations (% w/w) of ALM (a) 0.2 (b) 0.4 (c) 0.6 (d) 0.8 and (e) 1.0 and the % reflectance of carbonyl peak of ALM at 1650 cm^-1^.


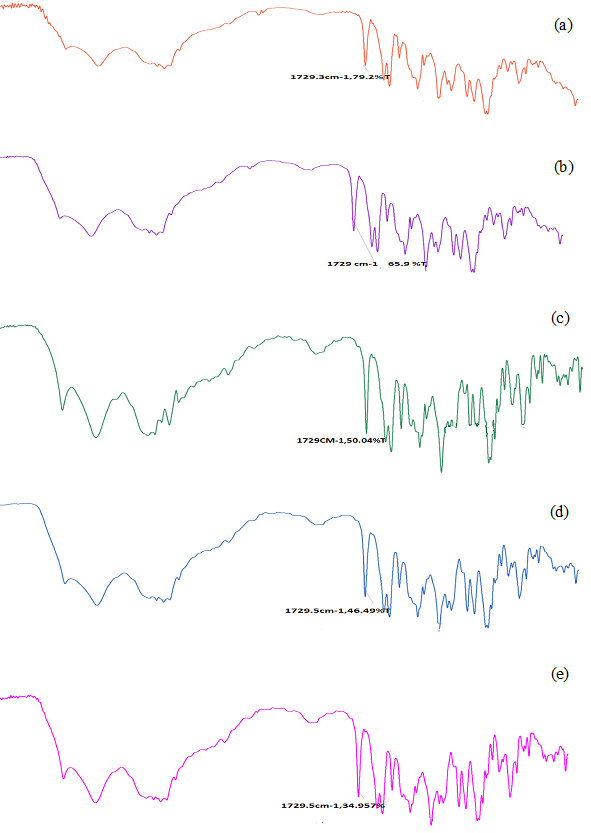


**Figure S1.** Transmittance spectra for various concentrations (% w/w) of DOX (a) 0.6 (b) 0.8 (c) 1.0 (d) 1.2 and (e) 1.4 and % transmittance of the carbonyl peak of DOX at 1729 cm^-1^


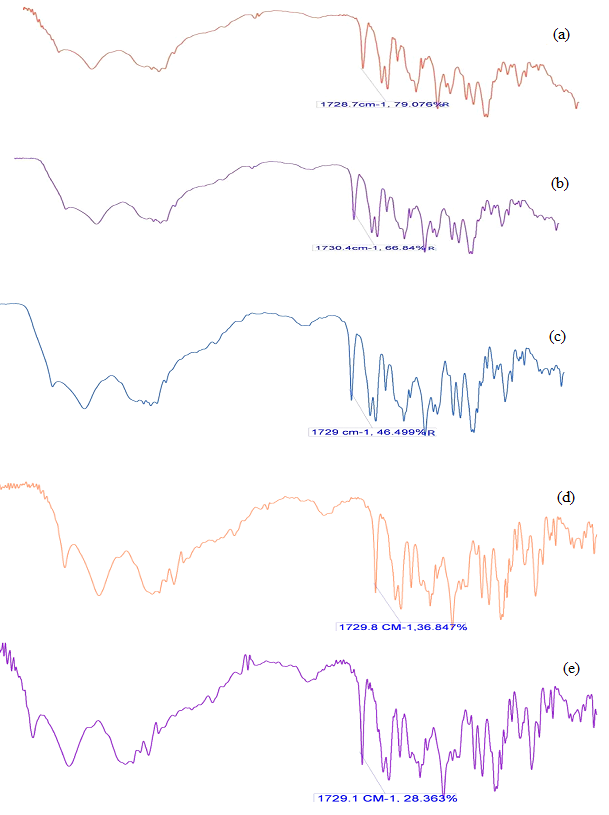


**Figure S2.** Reflectance spectra for various concentrations of DOX (% w/w) (a) 0.6 (b) 0.8 (c) 1.0 (d) 1.2 and (e) 1.4 and the % reflectance of carbonyl peak of DOX at 1729 cm^-1^.


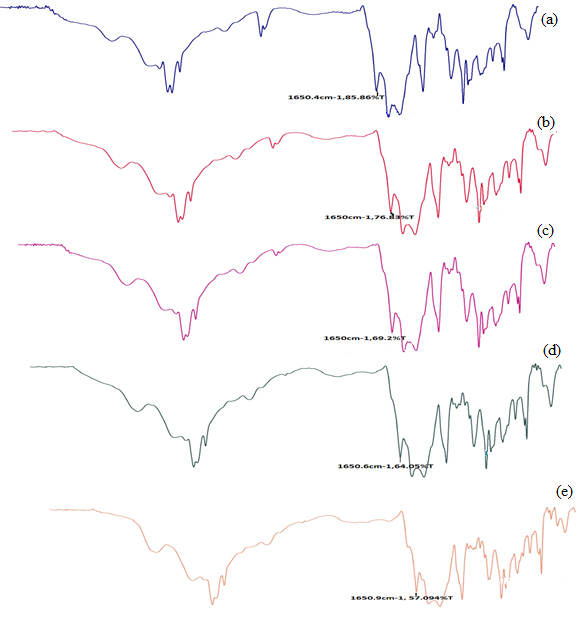


**Figure S3.** Transmittance spectra for various concentrations (% w/w) of ALM (a) 0.2 (b) 0.4 (c) 0.6 (d) 0.8 and (e) 1.0 and the % transmittance of carbonyl peak of ALM at 1650 cm^-1^.


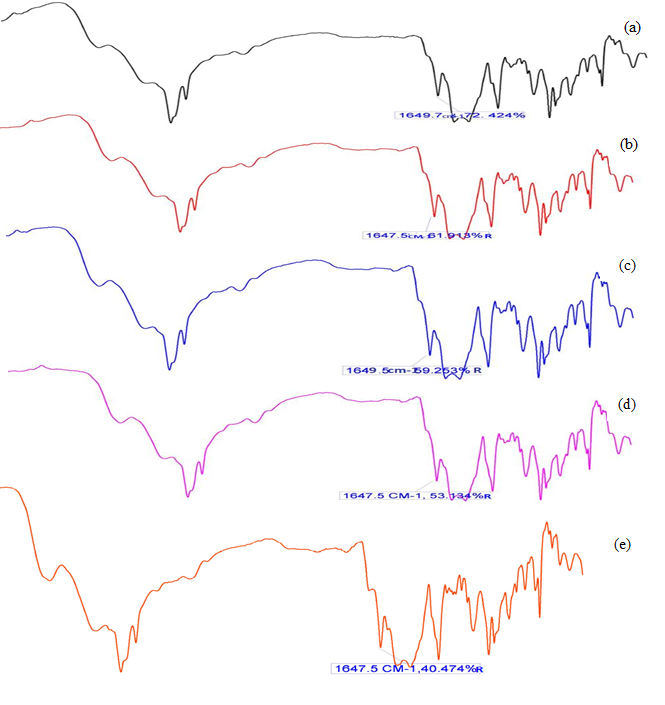


**Figure S4.** Reflectance spectra for various concentrations (% w/w) of ALM (a) 0.2 (b) 0.4 (c) 0.6 (d) 0.8 and (e) 1.0 and the % reflectance of carbonyl peak of ALM at 1650 cm^-1^.
